# Comparative performances analysis of neonatal ventilators

**DOI:** 10.1186/s13052-015-0112-z

**Published:** 2015-02-08

**Authors:** Ilaria Baldoli, Selene Tognarelli, Rosa T Scaramuzzo, Massimiliano Ciantelli, Francesca Cecchi, Marzia Gentile, Emilio Sigali, Paolo Ghirri, Antonio Boldrini, Arianna Menciassi, Cecilia Laschi, Armando Cuttano

**Affiliations:** The BioRobotics Institute, Scuola Superiore Sant’Anna, viale Rinaldo Piaggio,34, Pontedera, PI 56025 Italy; Centro di Formazione e Simulazione Neonatale “NINA”, U.O. Neonatologia, Azienda Ospedaliera Universitaria Pisana, via Roma 67, Pisa, 56126 Italy; Istituto di Scienze della Vita, Scuola Superiore Sant’Anna, piazza Martiri della Libertà 33, Pisa, 56100 Italy; University of Pisa, Pisa, Italy

**Keywords:** Mechanical ventilation, Neonatal pulmonary medicine, Respiratory technology

## Abstract

**Background:**

Mechanical ventilation is a therapeutic action for newborns with respiratory diseases but may have side effects. Correct equipment knowledge and training may limit human errors. We aimed to test different neonatal mechanical ventilators’ performances by an acquisition module (a commercial pressure sensor plus an isolated chamber and a dedicated software).

**Methods:**

The differences (ΔP) between peak pressure values and end-expiration pressure were investigated for each ventilator. We focused on discrepancies among measured and imposed pressure data. A statistical analysis was performed.

**Results:**

We investigated the measured/imposed ΔP relation. The ΔP do not reveal univocal trends related to ventilation setting parameters and the data distributions were non-Gaussian.

**Conclusions:**

Measured ΔP represent a significant parameter in newborns’ ventilation, due to the typical small volumes. The investigated ventilators showed different tendencies. Therefore, a deep specific knowledge of the intensive care devices is mandatory for caregivers to correctly exploit their operating principles.

## Background

Respiratory diseases are among the main causes of morbidity and mortality for preterm newborns and infants. A proper and focused mechanical ventilation can be decisive for the survival of such patients in some cases. Assisted ventilation of newborns remains a great challenge for technical staff, especially considering the wide variety of infants (e.g. weight varying from 500 g to 3–4 kg, so Vt varying from 3 to 24 ml). Based on this, the outcome of ventilation process is affected by the risk of side effects and complications, in particular because of the sensitivity of lung tissues and the smallness of volumes involved [1,2].

Given the complexity of the application domain, a continuous education program is necessary to train neonatologists and nurses, in order to give them adequate practical knowledge and experience to face hindrances.

High fidelity training is the best way to reach this aim, since it represents a completely interactive training system based on innovative strategies in a realistic clinical scenario [3-5].

In this framework, we are actively involved in a national research project (MERESSINA project, founded by the Italian national Commission for the Education and Training) about the design, development and testing of a neonatal pulmonary simulator able to represent lungs physiological features in a high fidelity model. In more detail, the simulator has been designed to reproduce infants’ breathing patterns, in both cases of controlled and assisted ventilation, and it is based on a multi-compartment model composed of five autonomous units replicating the anatomy of the human lobes [6,7].

In order to ensure the adaptability of the designed simulator to the wide range of ventilation conditions that can be set during a real training session, a study of the performances of different Intensive Care Units (ICUs) neonatal ventilators was carried out as similarly reported in the literature [8]. Our study was focused on the pressure values delivered at the Pressure Inspiratory Peak (PIP) and at the end of the expiratory phase - the Positive End Expiratory Pressure (PEEP), and in particular, on the pressure values difference (ΔP). ΔP is related to the tidal volume (V_T_), which expands lungs at each respiratory act, by lungs compliance (C, ml/cmH_2_O) according to eq. 1:

1 $$ {V}_T=C\Delta P=C\left(PIP- PEEP\right) $$

Being the control on ΔP, and hence on V_T_, a crucial feature of mechanical ventilation, especially considering the tiny lung volumes in newborn affected by pulmonary pathologies, neonatologists have to take great care of this aspect [9,10]: inaccuracies and errors in comparison to actual set values can appear paltry in an absolute sense, but they risk becoming significant if related to small volumes. V_T_ which results in less than the desired value can determine insufficient oxygenation, while an excessive volume can lead to stress and tissue damage.

In order to assess the correspondence between the imposed ΔP and the value measured downstream the ventilation circuit, acquisitions of the pressure signals delivered from different ICUs infant ventilators were performed with an appropriate experimental set up. In particular, the study was focused on the possible connection between delivered ΔP and other parameters of the controlled ventilation setting, e.g. inspiratory time and breathing frequency. We focused on pressure values because this parameter may be responsible for lung damage (so called barotrauma) in a volume-control ventilation modality, that is generally set on the modern neonatal ventilators.

## Methods

### Experimental set up

The tested medical devices are:

- n.3 Bear Cub 750 PSV Infant Ventilator (Bear Medical, Inc., CA, USA),
- n.1 Leoni Plus (Bomimed, Canada)
- n.1 Babylog 8000 Plus (Draeger Medical, Inc., USA).

The technical characteristics of the infant ventilators involved in the proposed analysis are reported (Table 1).

**Table 1:** **Major performances of 3 infant ventilators **

**Ventilator**	**Ventilation modalities**	**Time cycling**	**Flow cycling**	**Minimal pressure variation**	**Minimal flow variation**	**Loops and waves**	**Flow sensor calibration**	**Pressure working range**
Bear Cub 750 PSV	AC, AC-CF, SIMV/IMV, SIMV/PSV, SIMV-CF	YES	YES	1 cmH_2_O	0,5 L/min	YES	NO	0-72 cm H_2_O
Leoni Plus	IPPV/IMV, SIPPV,SIMV, PSV-SIPPV, PSV-SIMV	YES	YES	0,1 cmH_2_O	0.1 L/min	YES	YES	6-60 cmH_2_O
Babylog 8000 Plus	IPPV/IMV, SIPPV,SIMV, PSV	YES	YES	0,1 cmH_2_O	0,1 L/min	Only waves	YES	10-80 cm H_2_O

AC = Assisted Controlled, CF = Cycled Flow, SIMV = Synchronized Intermittent Mandatory Ventilation, IMV = Intermittent Mandatory Ventilation, PSV = Pressure Support Ventilation, SIPPV = Synchronized Intermittent Positive Pressure Ventilation, IPPV = Intermittent Positive Pressure Ventilation.

The same ventilation circuit was used for every device and it was adapted for making it compatible with a glass and sealed measurement chamber. The chamber was connected to the ventilation circuit thought an endotracheal tube (3.0 mm). During the tests we used 0.21 FiO_2_ and did not use the humidifier chamber.

**Figure 1:**
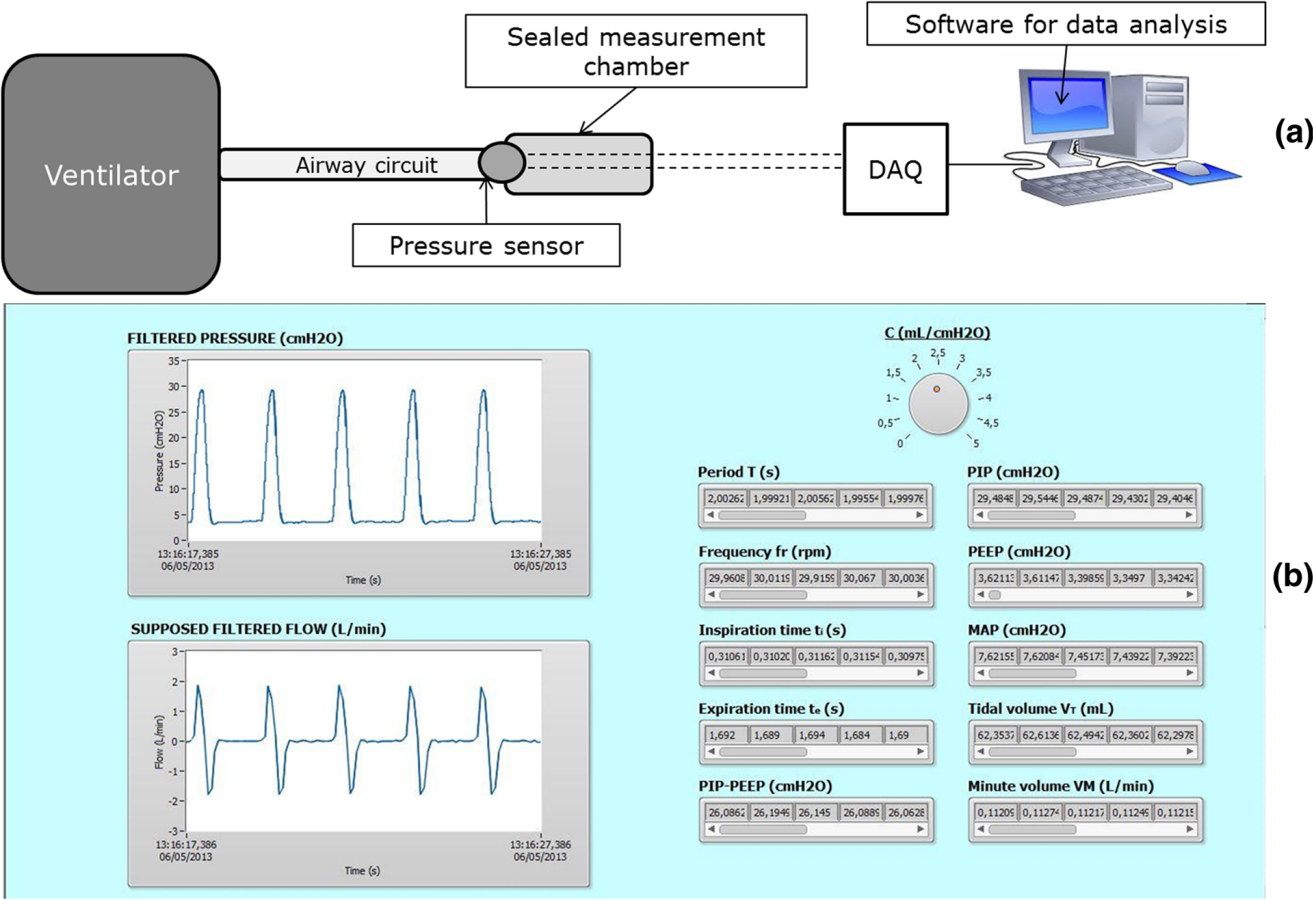
**Experimental set up. (a)** Simplified scheme of set up applied to test ventilators. **(b)** Labview GUI for extrapolating parameters from the pressure wave delivered from ventilators: on the left the acquired pressure curve is shown in real time and the related air flow -Q- is reported below, according to the fictional C range imposed by the user; on the right, the pressure wave features that are extrapolated by the software are shown: T , fr , t_i_ and t_e_ , PIP, PEEP and ΔP , MAP, supposed V_T_ and VM.

The pressure delivered by the ventilator was revealed inside the chamber thanks to an analogic pressure sensor (MS147105GT, Measurement Specialties, Hampton, USA) able to cover the measurement range of 0–34.5 kPa^a^ which results adequate for the required ventilator working range (equal to 0–5.5 kPa) based on physiological data of a preterm infant. Finally, the pressure signals were acquired by a data acquisition hardware (Multifunction DAQ System NI USB-6218, USA) and treated with a Labview (LabVIEW, NI, USA) software for amplification and filtering (Figure 1a). The same Labview software, equipped with a custom Graphic User Interface - GUI (Figure 1b), was employed to extrapolate the specific parameters of the pressure wave in the fully controlled modalities.

Pressure levels corresponding to imposed PIP and PEEP values were obtained searching for local maxima and minima of the calibrated function; the period (T) is the time distance between two sequent maxima. In order to obtain the inspiration time (t_i_), pressure signals were derived to obtain the flow curve and time intervals were detected from the positive segment of the flow wave.

Expiratory times (t_e_), respiratory frequency (fr) and mean airway pressure (MAP) are revealed according to eq.2 and eq.3:

2 $$ T={t}_i+{t}_e=60/{f}_r $$

3 $$ MAP=\frac{\left({t}_i*PIP\right)+\left({t}_e* PEEP\right)}{T} $$

Setting a fictional value for the C parameter (for newborn affected by neonatal respiratory distress syndrome, C varies from 0.5-1 cm H_2_O [10]), V_T_, flow trends (Q) and minute volume (VM) (eq.1, eq.4 and eq.5) are detectable for every respiratory period:

4 $$ Q=\frac{d{V}_T}{dt}=C\frac{d\left(PIP- PEEP\right)}{dt} $$

5 $$ VM={V}_T*fr $$

### Testing protocol

The pressure wave delivered from each infant ventilator was recorded. For each investigated device, the relation between measured ΔP (mΔP , mean ± SD) and imposed ΔP (iΔP) was investigated in two different working conditions: i) fixed fr, we varyed t_i_; ii) fixed t_i_ we imposed variation of fr. A critical analysis of the results, considering every combination of the parameters, was carried out.

Acquisition protocol was defined according to medical specifications: basal flow, inspiratory flow and PEEP values were fixed, varying PIP, fr and ti coefficients. Data acquisitions were performed according to the procedural settings described here:

Fixed parameters:

Basal flow: 10 L/min;

Inspiratory flow: 20 L/min;

PEEP: the minimum value reachable for each device (0 cmH_2_O for BEARs, 2.2 cmH_2_O for Leoni and 2.45 cmH_2_O for Babylog)

Variable parameters:

PIP: 10, 20, 30, 40 cmH_2_O

fr: 10, 50, 90 rpm

t_i_: 0.1, 0.3, 0.5, 0.9 s

Based on the clinical experience and considering the functional principles of the ventilators, some combinations of the chosen parameters are incompatible:

- by fixing t_i_ equal to 0.1 s, the maximum PIP value reachable by the ventilator is 20 cmH_2_O
- in case of 90 rpm, ti equal to 0.1 s and 0.3 s are the solely time values admissible in the procedure, being fr and t_i_ mathematically related by the inspiratory-expiratory times ratio (I:E) according to eq.6:

6 $$ fr=\frac{I:E}{\left(1+I:E\right)*{t}_i} $$

### Data analysis

**Figure 2:**
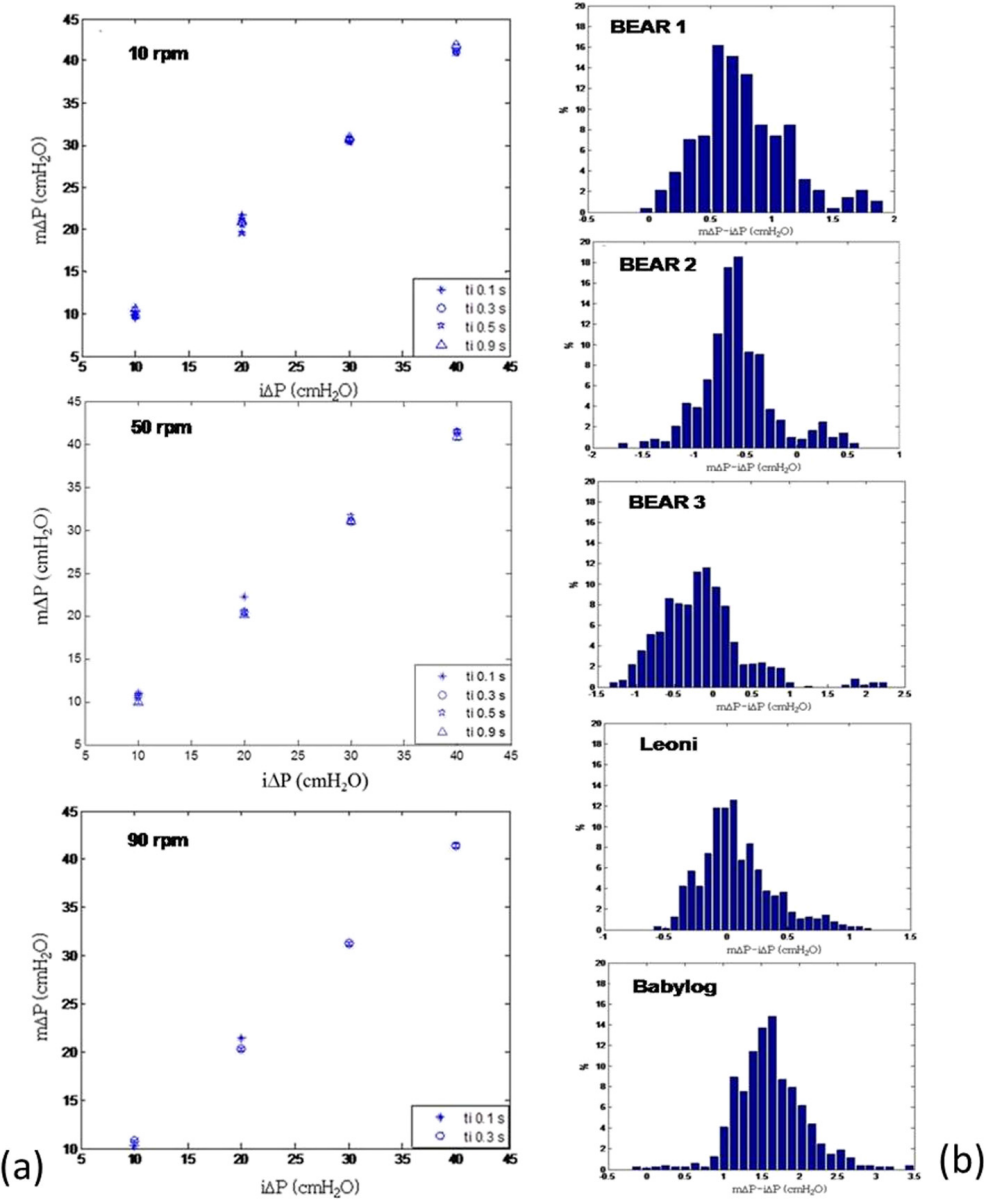
**ΔP experimental evaluation. (a)** Trend of measured ΔP vs imposed ΔP values by using the BEAR CUB n.1 ventilator by varying ti values. **(b)** Histograms of mΔP-iΔP distributions for the 3 ventilators (n.3 BEAR, n.1 Leoni, n.1 Babylog), by using setting parameters used in clinical practice.

Pressure wave, delivered for the 34 possible combinations of parameters described above, was acquired for three minutes for each ventilator. Thanks to the custom software, ΔP values were extrapolated and averaged (mean ± SD values were reported). Measured ΔP and comparison between the mean and the imposed values were related to the chosen ΔP by varying both fr and t_i_ (Figure 2a).

The distribution of the set of differences between measured and imposed ΔP was studied for each ventilator (Table 2). The study was then expanded to the selection of ΔP obtained just with settings owing to clinical practice. For the 3 Bear Cub ventilators, we focus on intra-device variability as well. Finally, statistical analysis of the data was carried out (Table 3 and Figure 2b).

**Table 2:** **Statistic features of mΔP - iΔP distributions for the 3 ICUs infant ventilators under investigation**

	**mean (mΔP - iΔP) (cmH**_**2**_ **O)**	**std dev (cmH**_**2**_ **O)**	**max (cmH**_**2**_ **O)**	**min (cmH**_**2**_ **O)**
LEONI	0,08	0,29	1,17	−0,57
BABYLOG	1,62	0,46	3,46	−0,13
BEAR	0,09	0,43	1,61	−1,03

Ideally, Mean and SD values should tend to 0.

**Table 3:** **Comparison among results from all mechanical ventilators, after the implemented tests (i.e. with settings owing to clinical practice)**

**Statistic features for the 3 ICUs infant ventilators**				
	**Mean (mΔP- iΔP) (cmH**_**2**_ **O)**	**SD (cmH**_**2**_ **O)**	**max (cmH**_**2**_ **O)**	**min (cmH**_**2**_ **O)**
LEONI	0,05	0,99	2,90	−6,59
BABYLOG	1,47	0,77	3,56	−3,30
BEAR	0,12	0,68	2,95	−1,83
**BEAR Ventilator: INTRA-VARIABILITY of the Results**				
BEAR n.1	0,78	0,77	3,18	−2,49
BEAR n.2	−0,26	0,71	2,19	−1,70
BEAR n.3	−0,17	0,56	3,46	−1,29

## Results

Each ventilator showed a markedly linear trend (R^2^ > 0.99) and there were no tendencies introduced by either fr or t_i_ (Figure 2a).

Differences between measured ΔP (mΔP) and imposed ΔP (iΔP), do not reveal univocal trends related to PIP, fr or ti. Basically, as reported in Figure 2a for the Bear Cub ventilator, the mΔP- iΔP value increases applying high PIP values in case of low ti (e.g. ti: 0,1 s), because such limited time is insufficient to practice the required pressure impulse.

mΔP- iΔP was studied for each ventilator, revealing the features resumed in Table 2.

The presence of significant divergences between mΔP and iΔP induced a further analysis, taking into account the results obtained in case of setting parameters usually employed in clinical practice. This choice allowed us to understand if such unexpected results, not entirely negligible, are related just to unusual settings.

In particular, the detection of optimal functioning parameters range had been possible based on guidelines of medical practice [10]:

- 5 ≤ imposed ΔP ≤ 30 cmH_2_O;
- ti range: 0.3 - 0.5 s;
- I:E = 1:2–1:3.

The last two conditions imply 40 rpm as minimal fr (considering the worst working conditions: ti equal to 0.3 s and I:E equal to 1:2) and 100 rpm as maximal fr (ti: 0.5 s and I:E:1:3). Based on this, data acquisitions were carried out by imposing:

fr: 50 rpm, ti: 0.3 and 0.5 s;

fr: 90 rpm and ti: 0.3 s.

In these working conditions, the parameters derived from data analysis are clearly improved (Table 3).

For each ventilator (n.3 BEAR, n.1 Leoni, n.1 Babylog), the set of the selected differences is presented in histograms (Figure 2b) and their distribution was studied with the D’Agostino-Pearson normality test, revealing, in each case, a markedly non-Gaussian behavior (P < 0.01).

Since sampling distributions are quite numerous (284 at least), original data populations are likely Non-Gaussian distributions, therefore a comparison among the five samples was carried out with the non-parametric Kruskall-Wallis test. This analysis revealed some sets have significantly different results. (P < 0.01).

The Kruskall-Wallis test was used also to compare data from the 3 BEAR CUB ventilators, showing significant divergences (P < 0.01). In case of difference, we performed Dunn’s test using multiple, stepdown comparisons (Kruskall-Wallis analysis): each couple of ventilator differs significantly (P < 0.01) from the other one, also from the couples composed of devices of the same brand.

## Discussion

We tested the performances of three most largely used ICUs infant ventilators in Italy and Europe, by using a simple testing workbench. For the Bear Cub 750 PSV Infant Ventilator, three devices were tested in order to investigate the performances of different ventilators of the same brand and to underline the intra-variability of the results. This choice allowed us to compare not only devices of different brands, but also ventilators of the same trademark.

The working ranges of the parameters were intentionally chosen wider in comparison to the ones actually employed in clinical practice in order to test the ventilator performances at the working limits, which are rarely used into the NICUs, but still guaranteed by the head offices. Moreover, being a comparative study about the ventilator performances with the ultimate goal to design and develop an innovative simulator for medical training, we need to replicate the entire range of operation, to allow us to fully investigate the consequences of extreme choices during mechanical ventilation. In more detail, the imposed flows are higher than values employed in clinical practice because they allow reaching desired PIP for every device.

Pressure data show a relevant discrepancy between peak values set on the ventilators and the measured ones. These differences become even larger when setting extreme ventilation parameters (i.e. ΔP values of −6.59 or +3.56 cmH_2_O). On the contrary, in conditions more similar to physiological settings, such differences tend to be reduced. Minimum discrepancies are negligible in children and adult patients, but may be important in newborns. Indeed, extremely low birth weight preterms need very small V_T_ (e.g. a newborn having a weight of 500 g requires a 2–3 ml gas exchange volume + 2.5 ml dead space) and it is possible that even small changes in the PIP can affect delivered volume. For instance, with a given compliance of 1 ml/cmH_2_O the discrepancy of 6 cmH_2_O between set PIP and measured PIP causes a variation of 6 ml in V_T_. Considering a 2000-grams-weightened newborn, who has a theoretical tidal volume of 10 ml, the variation is more than 50% of the desired value.

Moreover, we cannot exclude that also in non-conventional ventilation techniques, such as in volume-target ventilation, differences between set volumes and delivered volumes could occur. Consequently, developing lungs can be damaged by excess of volume and/or pressure, since acceptable values range is actually small. Indeed, it is well known that injury induced by mechanical ventilation is a major co-factor of BPD.

Our study was carried out in optimal ventilation conditions, hardly reproducible in vivo, for example, no losses through endotracheal tube, no secretion, compliance and resistance being constant during each single breath. Therefore, it is possible that during a real ventilation of infants, which involves all the variables mentioned above, differences may be even higher.

A limitation of our study can be due to the ventilators age: in fact, the Bear and Babylog are older than 15 years. Anyway, some discrepancies, even if less important (basing on the mean (mΔP- iΔP) value), have been also found with the Leoni plus ventilator, that is about 2 years old.

Finally, we have to underline that measures among ventilators of the same brand can vary.

For all these reasons, it is mandatory to have adequate education and a correct knowledge of the equipment, in order to predict and limit the margin of error during mechanical ventilation and to minimize the possible iatrogenic damage to newborns.

It is worth to mention that in our opinion the knowledge about the accuracy limitations of commercial ventilators could be very important during a simulation program. However, our study was an only in vitro analysis, and additional surveys about the benefit for training sessions will be further investigated.

In conclusions, we analyzed three different ICUs neonatal ventilators performances, comparing inter- and intra-devices variations. We focused on the difference pressure values (ΔP) between the inspiration peak data and the pressure delivered at end of the expiratory phase. Indeed, ΔP is one of the most important features of ventilation modalities because it is related to the V_T_, which is responsible for lungs expansion at every respiratory act.

It has to be specified that the Bear Cub ventilators measure airway pressure at the patient connection while the Draeger ventilators (Babylog) use internal inspiratory and expiratory pressure sensors to compute airway pressure based in the known pressure drop in the patient circuit. Even if it is not a very plausible hypothesis since the ventilation circuit is closed, we cannot certainly exclude that this aspect could account for the different performances of the ventilators found in our study.

Our study underlines that the pressure differences reported represent a negligible discrepancy for children and adult patients, but they may be significant in newborns, due to the small volumes involved. In addition, during a real ventilation procedure, the optimal working conditions used in the analysis are not easily reproducible; therefore, these differences may be even higher.

Based on these, even if in clinical practice the use of Vt monitoring, the use of optimal PCO_2_ and PO_2_ target values, and the transcutaneous PCO_2_ and PO_2_ monitoring should guide the ventilator management of the more vulnerable infants, nevertheless staff are required to get a correct and deep knowledge also of the equipment and to undergo adequate training, in order to limit the margin of error during mechanical ventilation and minimize the induced damages to newborns’ lungs.

## Endnote

^a^34,5 kPa = 351.5 cm H_2_O.

## References

[1] Moretti C, Papoff P. Lung development and pulmonary malformations. In: Buonocore G, Bracci R, Weindling M. Neonatology – a practical approach to neonatal management. 1st ed. Springer-Verlag. 2012.

[2] Martin RJ, Fanaroff AA, Walsh MC. Fanaroff and Martin’s Neonatal-Perinatal Medicine: Diseases of the Fetus and Infant-Expert Consult. 9th edn. Mosby.

[3] McGaghie WC, Siddall VJ, Mazmanian PE, Myers J (2009). Lessons for Continuing Medical Education From Simulation Research in Undergraduate and Graduate Medical Education Effectiveness of Continuing Medical Education: American College of Chest Physicians Evidence-Based Educational Guidelines. Chest.

[4] Curtis MT, Diaz Granados D, Feldman M (2012). Judicious use of simulation technology in continuing medical education. J Contin Educ Health Prof.

[5] Flechelles O, Ho A, Hernert P, Emeriaud G, Zaglam N, Cheriet F, Jouvet PA (2013). Simulations for mechanical ventilation in children: review and future prospects. Crit Care Res Pract.

[6] Baldoli I, Tognarelli S, Cecchi F, Scaramuzzo RT, Ciantelli M, Gentile M. An Active One-Lobe Pulmonary Simulator with Compliance Control for Medical Training in Neonatal Mechanical Ventilation. J Clin Monit Comput. 2013. [Epub ahead of print] 10.1007/s10877-013-9521-y 24126618

[7] Scaramuzzo RT, Ciantelli M, Baldoli I, Bellanti L, Gentile M, Cecchi F (2013). MEchatronic REspiratory System SImulator for Neonatal Applications (MERESSINA) project: a novel bioengineering goal. Med Devices (Auckl).

[8] Abbasi S, Sivieri E, Roberts R, Kirpalani H (2012). Accuracy of tidal volume, compliance, and resistance measurements on neonatal ventilator displays: an in vitro assessment. Pediatr Crit Care Med.

[9] Morley CJ. Treatment of respiratory failure: mechanical ventilation. In: Buonocore G, Bracci R, Weindling M. Neonatology – a practical approach to neonatal management.1st edn. Springer-Verlag 2012;497–507.

[10] Waldemar AC, Di Fiore JM. The respiratory system: assessment of pulmonary function. 1st edn. Mosby. 2010;1092-1104.

